# Cytokines, Genetic Lesions and Signaling Pathways in Anaplastic Large Cell Lymphomas

**DOI:** 10.3390/cancers13174256

**Published:** 2021-08-24

**Authors:** Jean-Philippe Merlio, Marshall E. Kadin

**Affiliations:** 1 Tumor Biology and Tumor Bank Laboratory, Centre Hospitalier et Universitaire de Bordeaux, 33600 Pessac, France; 2 INSERM U1053, University Bordeaux, 33000 Bordeaux, France; 3 Department of Pathology and Laboratory Medicine, Brown University Alpert School of Medicine, Providence, RI 02903, USA; 4 Department of Dermatology, Boston University, Boston, MA 02215, USA

**Keywords:** anaplastic large cell lymphoma, oncogenesis, cytokine, signaling pathways

## Abstract

**Simple Summary:**

This review summarizes the main features of anaplastic large cell lymphoma (ALCL) subtypes focusing on activated biological pathways that may have clinical significance for diagnosis, prognosis and personalized therapy.

**Abstract:**

ALCL is a tumor of activated T cells and possibly innate lymphoid cells with several subtypes according to clinical presentation and genetic lesions. On one hand, the expression of transcription factors and cytokine receptors triggers signaling pathways. On the other hand, ALCL tumor cells also produce many proteins including chemokines, cytokines and growth factors that affect patient symptoms. Examples are accumulation of granulocytes stimulated by IL-8, IL-17, IL-9 and IL-13; epidermal hyperplasia and psoriasis-like skin lesions due to IL-22; and fever and weight loss in response to IL-6 and IFN-γ. In this review, we focus on the biology of the main ALCL subtypes as the identification of signaling pathways and ALCL-derived cytokines offers opportunities for targeted therapies.

## 1. Main ALCL Subtypes

The different ALCLs represent about 16% of peripheral T cell lymphomas [1]. As summarized in Table 1, they form a heterogeneous group of CD30-positive T cell non-Hodgkin lymphomas according to their site of onset (systemic, cutaneous or breast implant-associated) and their genetic features, with several groups according to the presence of ALK rearrangement and subsequent ALK expression defining ALK-positive or ALK-negative ALCLs [2,3,4]. ALK-negative ALCL may carry DUSP22 rearrangements and/or TP63 rearrangements. ALK-positive and DUSP22-rearranged ALCLs have a better prognosis than triple-negative ALCL and TP63-rearranged ALCL, which have the worst outcome [2,3,5,6,7]. The site of origin is critical as illustrated by cutaneous ALCL (C-ALCL), which is commonly ALK-negative [8,9,10]. C-ALCL has a favorable prognosis with a 5-year disease specific survival of 90%, even in the presence of regional lymph node involvement or putative adverse genetic lesions such as the rare *TP63* rearrangement [3,11,12].

### 1.1. Systemic ALK+ ALCL

**Figure 1:**
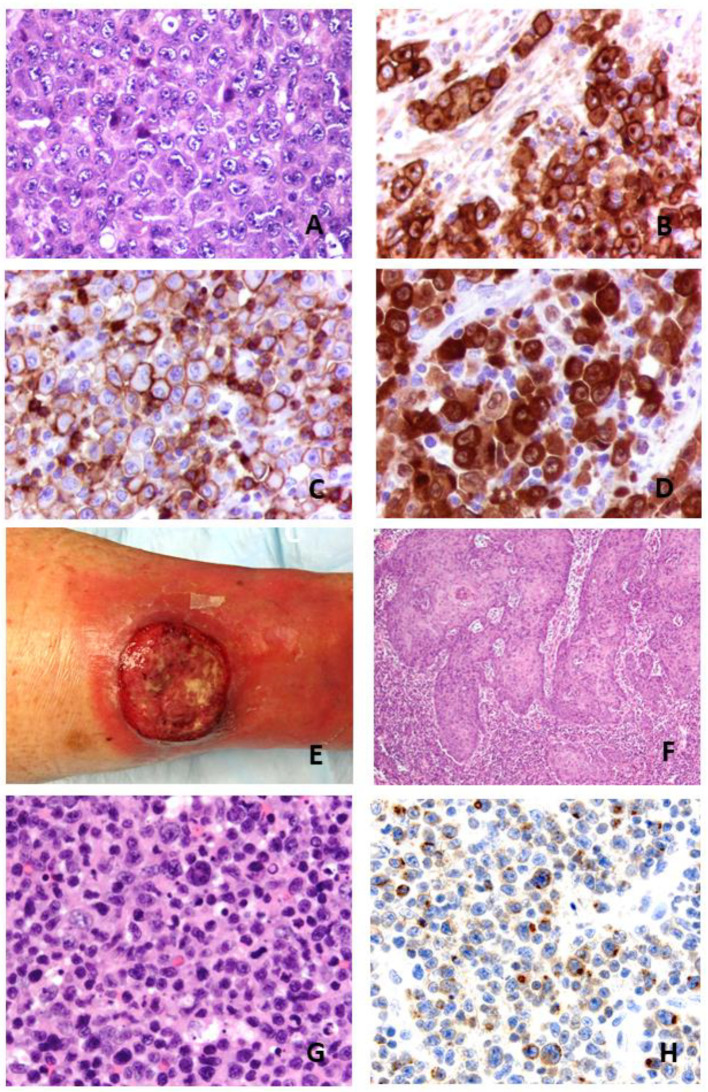
Morphological features of systemic ALK+ anaplastic large cell lymphoma. (**A**): HE: hematein and eosin staining ×400. Common morphological variant with hallmark cells. (**B**–**D**): CD30, EMA and ALK immunostainings (×400). Courtesy of Dr Marie Parrens, CHU de Bordeaux, Pessac, France. Clinical and pathological features of a cutaneous anaplastic large cell lymphoma. (**E**): Tumor of lower leg. (**F**): Pseudoepitheliomatous hyperplasia on HE stain (×200). (**G**): Cytomorphology on HE stain (×400). (**H**): IL-17A immunostain of tumor cells (×400).

Systemic ALK+ ALCL mainly occurs in children and young adults with a male predominance, affecting the lymph nodes as well as extranodal sites (Table 1). ALK+ ALCL presents with several morphological variants sharing the hallmark anaplastic cells (common, small cell, lymphohistiocytic, Hodgkin-like or composite), extensively reviewed elsewhere [4,13]. Tumor cells strongly express CD30, EMA, CD25, BCL6 and cytotoxic molecules such as TIA1, granzyme B and perforin (Figure 1). The loss of several T cell markers including CD3, CD2, CD5, CD7 and the T cell antigen receptor (TCR) is a common feature of ALK+ ALCL cells, leading to an apparent “null cell” phenotype that may be used for the differential diagnosis with other CD30+ PTCL or CD30+ mycosis fungoides with large cell transformation [4,13,14]. ALK is localized at the nuclear/nucleolar or cytoplasmic level according to the partner gene involved in the fusion transcripts [15]. Although NPM1/ALK fusion transcripts are the most common, the other *ALK* partners are *TPM3* (1q25), *ATIC* (2q35), *TFG* (3q21), *TPM4* (19p13.1), *MYH9* (22q11.2), *RNF213* (17q25), *TRAF1* (9q33.2), *CLTC* (17q23) and *MSN* (Xq11) [4,16,17,18]. The identification of the partner gene thus far has little impact on patient management, and ALK immunostaining is sufficient for diagnosis [14]. Monitoring of minimal residual disease by the detection of *ALK* transcripts has been mainly used in pediatric protocols and was recently implemented by a digital PCR technique based on a universal 3′*ALK* probe [16,19,20].

### 1.2. Systemic ALK- ALCL

Systemic ALK- ALCL frequently occurs in older patients and involves the lymph nodes and extranodal tissues, with a more aggressive course, B symptoms and advanced stages [21]. Absence of ALK expression combined with various morphologies may constitute a diagnostic challenge for differential diagnosis with either CD30+ PTCL or classic Hodgkin lymphoma (CHL) [1,4,14]. The key features are the cohesive pattern with sinus infiltration, the presence of hallmark cells, abundant cytoplasm, strong and uniform expression of CD30, frequent loss of T cell markers and expression of cytotoxic molecules (TIA1, granzyme B and perforin) or EMA. In contrast to CHL, ALK- ALCL does not express PAX5, EBV transcripts or LMP1 [4,18].

Genomic profiling of 32 clinical samples and 5 ALCL cell lines by microarray analysis identified overexpression of the BCL6, PTPN12, CEBPB and SERPINA1 genes in ALK-positive ALCL, whereas the CCR7, CNTFR, IL-22 and IL-21 genes were overexpressed in ALK-negative ALCL. High levels of interferon regulatory factor 4 (IRF4), which induces MYC expression, were observed in all ALCL subtypes [22]. Decreased expression of MYC-associated factor X (MAX), identified in both ALK+ and ALK- ALCLs, was associated with an uncommon morphology and expression of MYC and cytotoxic molecules in patients with adverse prognosis [23]. Others detected a high expression of MYC in 37% of ALK+ ALCLs, with a common morphology in older patients with a shorter survival [24]. 

Genomic profiling showed that ALK+ ALCL displays a homogeneous cytotoxic/Th1 signature in contrast to ALK- ALCL that has either a cytotoxic profile or a Th2-associated signature. The TH2 group was enriched in cases displaying a *DUSP22* rearrangement [25]. The presence of *TP63* rearrangement in 12% of ALK- ALCLs defines a third ALCL category [2]. The different rearrangements such as inv(3)(q26;q28) or t(3;6)(q28;p22.3) generate *TBL1XR1-TP63* or *TP63-ATXN1* fusion transcripts, leading to expression of a dominant negative oncogenic p63 isoform [26]. In systemic ALK- ALCL, *TP63* rearrangement is associated with a poorer outcome. Exceptional C-ALCLs displaying both DUSP22 and TP63 rearrangements have been reported [2,3,7]. The absence of either *ALK*, *DUSP22* or *TP63* rearrangement defines a “triple-negative” subset of ALK- ALCL with a 5-year OS rate of 42% [2].

### 1.3. Cutaneous ALCL

Cutaneous ALCL belongs to primary cutaneous CD30+ lymphoproliferative disorders together with lymphomatoid papulosis and borderline cases. C-ALCL is defined by the presence of more than 75% of large cells expressing CD30+ in patients without evidence or history of epidermotropic T cell lymphoma [12,27]. The disease is limited to the skin, affecting the trunk, face and extremities (Figure 1). Regional lymph node involvement may exist but does not affect prognosis [11]. Most cases express a CD4+ T cell phenotype with loss of CD2, CD3 or CD5 and frequent expression of cytotoxic proteins [28]. However, some cases are CD4-/CD8+ or CD4+/CD8+. Unlike systemic ALCL, C-ALCLs usually express CLA and CD158k but do not express EMA. C-ALCLs also express skin-homing molecules such as CCR4, CCR10 and CCR8. MUM1 encoded by the *IRF4* gene is strongly positive, and CD15 is expressed in about half of the cases [10,27]. 

C-ALCLs exhibit some privileged cytogenetic features with a *DUSP22* rearrangement in up to 30% of cases [5,6]. *DUSP22*-rearranged ALCLs not only arise at extranodal sites but also present with sheets of large hallmark cells admixed with smaller cells having both nuclear indentations sometimes giving rise to doughnut cells [2,5,29,30]. The phenotype is non-cytotoxic with absence of EMA expression [5,30]. Interestingly, expression of the lymphoid enhancing transcription factor (LEF1) predicts *DUSP22* rearrangement with high specificity and sensitivity [31]. Some C-ALCLs and other CD30+ lymphoproliferations were shown to harbor an *NPM1-TYK2* fusion gene, leading to STAT1/3/5 activation [32]. However, common TYK2 expression in ALCL cannot serve to identify such cases. Very few C-ALCLs were found to display a *TP63* rearrangement that does not affect their good prognosis [3,7].

### 1.4. Breast Implant-Associated Anaplastic Large Cell Lymphoma (BIA-ALCL)

This rare disease, generally occurring 8–10 years after implantation, presents with either a peri-implant seroma elicited by chronic inflammation or a capsular invasion, sometimes as a tumor. It shares many features with ALK-ALCL including expression of EMA, cytotoxic proteins and phospho-STAT3 [33]. CD30+ immunostaining and T cell monoclonality are diagnostic criteria. In late seromas generated by infection or implant rupture, high levels of IL-10, IL-13 and Eotaxin and an elevated IL10/IL-6 ratio are features of the effusion milieu [34]. BIA-ALCL exhibits Th-2 differentiation as neoplastic cells express GATA3 and FOXP3 [34] Nevertheless, bacterial biofilm infection of implants has been implicated in provoking chronic inflammation as a trigger for BIA-ALCL [35].

## 2. ALCL Oncogenesis

### 2.1. ALCL Cell of Origin

The detection of NPM-ALK transcripts in neonatal cord blood suggested that ALK+ cells may originate from either stem cells or early thymic progenitors (ETP) [36]. A transgenic mouse model with expression of NPM-ALK under the CD4+ promoter indicated that ALK+ ALCL could emerge from ETP. CD4/NPM-ALK expression functioning as a surrogate TCR permits cells to bypass thymic selection through upregulation of Notch1 expression [37]. However, malignant transformation of CD4+ lymphocytes by NPM-ALK also suggests that ALK+ ALCL may derive from rare peripheral mature CD4+CD30+ T cells [38]. Transduction of NPM-ALK into mature CD4+ peripheral T cells after CD3/CD28 co-stimulation produced NPM-ALK-transformed lymphocytes displaying an early thymic precursor gene expression signature [39]. NPM-ALK induced lymphocyte survival and spreading but also reversed the mature T cell phenotype into an ETP phenotype associated with expression of pluripotency-associated transcription factors such as OCT4, SOX2 and NANOG under the control of HIF2A [39]. HIF2A silencing abrogated NPM-ALK and STAT3 activity, suppressing the cell growth of NPM-ALK-transformed CD4+ lymphocytes. Whether the above transgenic mice or preclinical models reflect a real difference between pediatric and adult ALCL is still a matter of debate. In a subset of ALCL (14%), a germline TCR also supports lack of thymic maturation [4,16,37].

### 2.2. ALK+ ALCL

The hallmark of ALCLs is the expression of the CD30 antigen, a member of the tumor necrosis factor (TNF) receptor superfamily normally expressed in peripheral T cells after antigen stimulation of the CD3/TCR complex [40,41]. Malignant transformation of CD4+ lymphocytes by NPM-ALK was shown to mimic physiological cytokine signals and/or TCR triggering [37,38,42]. The prototype of ALK+ ALCL is associated with NPM1-ALK fusion transcripts resulting from t(2;5)(p23.2;q35.1) translocation [43]. In ALK+ ALCL, the N-terminal domain of the partner activates the catalytic domain of the ALK protein through homo- and hetero-dimerization [4,16]. NPM-ALK triggers several intracellular signaling pathways involving PLCgamma, PI3K-AKT, Ras ERK, JAK3-STAT3 and STAT5 [13,17,44]. The MAPK-ERK pathway is mostly associated with proliferative effects, whereas the JAK3-STAT3 pathway and the PI3K-AKT pathway promote cell survival and phenotypic changes (Table 2) [42].

### 2.3. STAT3 a Pivotal Transcription Factor in Most ALCL Subtypes

**Figure 2:**
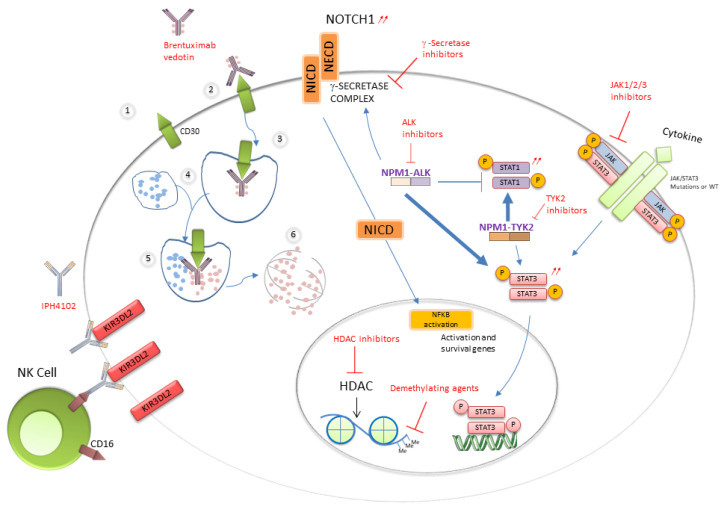
Activated pathways and therapeutic targets in ALCLs.

Many of the biological features of ALK+ ALCL result from epigenetic deregulation triggered by STAT3 activation. This applies to both ALK+ and ALK- ALCL, except for the ALK- ALCL subtype bearing *DUSP22* rearrangement [5,45]. NPM-ALK triggers STAT1 phosphorylation and degradation and promotes STAT3 upregulation. STAT3 also induces the expression of DNA methyl transferase 1 that promotes epigenetic reprogramming of ALK+ cells that frequently lack expression of CD3+, TCRs and related molecules, including CD3ε, zeta-chain-associated protein kinase 70 (ZAP70), linker for activation of T cells (LAT) and lymphocyte cytosolic protein 2 (LCP2) [46]. Then, STAT3 induces immune escape mediated by expression of TGF-beta, IL-10, ICOS and PDL1 (Figure 2). NPM-ALK, through binding of STAT3, was shown to promote Notch1 deregulation which can be inhibited by γ-secretase inhibitors (GSIs), leading to apoptosis [47]. In ALK+ ALCL, several miRNAs including three members of the miR-17-92 clusters are aberrantly overexpressed, while miR-155 is >10-fold overexpressed in ALK- ALCL [48]. Alternatively, other miRNAs such as miR-101, miR-29c and miR-26 are down-regulated in ALK+ and ALK- ALCL cell lines and primary human samples [48]. MiR-29a down-regulation is also driven by NPM-ALK activity and contributes to apoptosis blockade through epigenetic deregulation of MCL-1 expression [49]. A recent exome sequencing study also underscored the interplay between the STAT3 and Notch pathways in both ALK+ and ALK- ALCLs. A point mutation of T349P *NOTCH* was detected in 12% of ALK+ and ALK- ALCL patient samples [47].

STAT3 activation occurs in ALK- ALCLs through different mechanisms (Figure 2). Activating point mutations of *STAT3* and/or *JAK1* have been identified in 18% of nodal ALK- ALCLs and in 5% of C-ALCLs [50]. Another study of PTCL identified in ALK- ALCLs the highest rate of *STAT3* mutations (38%) that may combine with *JAK* mutations (15%), while some ALK+ ALCLs (13%) presented *STAT3* mutation alone [51]. The highest pY-STAT3 phosphorylation level is present in ALK- ALCL displaying a typical CD3-CD5- CD7- CD30+ phenotype [51]. Whatever the *JAK* gene status, ALK- cells are addicted to cytokine receptor signaling, and JAK inhibitor sensitivity correlates with STAT3 phosphorylation [52]. About 60% of BIA-ALCLs have mutations in at least one member of the JAK/STAT pathway including STAT3, JAK1 and STAT5B and in negative regulators such as SOCS3, SOCS1 and PTPN1 [53]. In addition, a majority of BIA-ALCL cases (74%) displayed recurrent mutations of epigenetic modifiers such as *KMT2C, KMT2D*, *CHD2* and *CREBBP* [53]. In rare ALK- ALCL subsets, chromosomal rearrangements creating chimeras combining a transcription factor (NFkB2 or NCOR2) with a tyrosine kinase (ROS1 or TYK2) were found to elicit STAT3 phosphorylation independent of JAK1 or STAT3 mutations [50].

### 2.4. STAT3-Independent ALK- ALCLs

ALK- ALCLs with 6p25.3 rearrangement are associated with *DUSP22* silencing and conserved IRF4 expression, while inactivation of the second allele by *DUSP22* mutation is uncommon [54]. Interestingly, *DUSP22*-rearranged ALCL is characterized by a unique global DNA demethylation profile associated with lack of STAT3 activation and overexpression of CCR8, HAND1, a developmental transcription factor and a group of cancer/testis-associated proteins. This profile includes upregulation of different costimulatory CD58 and HLA class II molecules and down-regulation of PD-1 that may contribute to the good prognosis of *DUSP22*-rearranged ALCL [55]. Among PTCL, recurrent mutations of the musculin gene (*MSC*^E116K^) encoding a basic helix-loop-helix transcription factor were found to be specific for 35% of *DUSP22*-rearranged ALCLs [45]. The dominant negative MSC^E116K^ protein promotes the growth of normal and neoplastic T cells by blocking the expression of the cell cycle inhibitor E2F2. This leads to upregulation of the CD30–IRF4–MYC axis in an autocrine feedback loop and confers susceptibility to the BET inhibitor JQ1 [45]. About 24% of ALK- ALCLs are characterized by ectopic co-expression of truncated *ERBB4* transcripts and *COL29A1* transcripts [56]. Such ERBB4-positive ALCLs frequently display a Hodgkin-like morphology and express MMP9. Two oncogenic truncated *ERBB4* transcripts arise from an intronic transcription start site and promote tumorigenesis partially blocked by the pan-HER inhibitor neratinib in experimental models [56].

## 3. Cytokines in ALCLs

### 3.1. Detection and Monitoring of Cytokines

CD30/TNFRSF8 is a co-stimulatory molecule expressed on activated T and B cells commonly used as a marker for neoplastic cells of cHL, systemic ALCL and CD30+ CLPD [41,57]. Serum levels of soluble CD30 (sCD30) correlate with tumor burden and normalize following successful treatment of cHL and ALCL [58,59]. Similarly, serum levels of the soluble truncated γ-chain of the IL-2 receptor (sCD25) expressed by activated immune cells correlate with disease activity and prognosis in HL [58,59,60] and mycosis fungoides (MF) [61]. Hanson et al. adapted a commercially available enzyme-linked immunoassay (ELISA) (R&D Systems, Minneapolis, MN, USA) to measure sCD30 in malignant and benign seromas and plasma of patients with breast implants [62]. sCD30 could be detected at concentrations of >1800 pg/mL in seromas of nine patients with BIA-ALCL but not in their plasma or serum and in none of the seven patients with non-neoplastic effusions [62]. 

Serum CD30 levels were prognostically significant in 116 patients with CD30+ CLPD, including CALCL, and 96 patients with early mycosis fungoides (MF) followed up to 20 years [63]. A significant positive correlation was found between sCD30 levels and sCD25, CD40L, IL-6 and IL-8. CD30+ CLPD-derived cell lines secrete sCD30, sCD25, IL-6 and IL-8. CD30+ CLPD patients with above normal sCD30 and sCD25 levels had worse overall and disease-related survivals. High sCD30 also identified patients with worse survival in early MF. Increased IL-6 and IL-8 levels correlated with poor disease-related survival in CD30+ CLPD patients [63]. 

### 3.2. Cytokines in ALK+ ALCL

Savan et al. analyzed circulating cytokine levels in ALK+ ALCL patients and detected elevated levels of IL-22, IL-17 and IL-8 in untreated patient samples. IL-22 and IL-17 were undetectable in all patients who were in complete remission after chemotherapy [64]. Knörr et al. analyzed sera of 119 uniformly treated pediatric ALK+ ALCL patients and 15 patients in remission, while 11 low-stage B cell lymphoma patients served as controls [65]. Concentrations of IL-9, IL-10, IL-17A, hepatocyte growth factor (HGF), sIL-2R and sCD30 were significantly elevated in initial sera of ALCL patients when compared to control groups, indicating an ALCL-type cytokine signature. Levels of IL-6, IFN-γ, IP- 10 and sIL-2R correlated with the stage, initial general condition, minimal disseminated disease, ALK antibody titers and risk of relapse among ALK+ ALCL patients. Only IL-6 showed an independent prognostic value in multivariate analyses [65]. Aberrant upregulation of interleukin 10 receptor subunit alpha (IL10RA) is observed in both ALK+ and ALK- ALCL and triggers STAT3 phosphorylation independently of NPM-ALK1 in ALK+ ALCL [66]. 

### 3.3. Signaling through IL-2R Activates the JAK/STAT Pathway in Cutaneous ALCL

Activation of JAK/STAT proteins was found to be involved in the signal transduction pathway mediated by the receptor for interleukin 2 in malignant T lymphocytes derived from cutaneous ALCL and Sézary syndrome [67]. Interaction of cytokine receptors such as IL-2R with their ligands induces activation of intracellular tyrosine kinases [68]. The high-affinity IL-2R is composed of three chains: α which is specific for IL-2, β and common γ. The cytoplasmic domain of the γ chain is associated with the tyrosine kinase Jak3, whereas the β chain is associated with Jak1 [69]. Binding of IL-2 to IL-2R results in the tyrosine phosphorylation of several substrates, including Jak3 and Jak l themselves, as well as the IL-2R β and γ chains [69]. Soluble IL-2 receptor (sIL-2R) also correlated with tumor burden in SCID mice xenografted with the ALCL line JB6 and was detected in the urine of JB6-transplanted mice [70]. 

### 3.4. Cytokines in BIA-ALCL

In contrast to ALK+ ALCL, neither IL-17A nor IL-17F was detected in 48 h cell cultures of established BIA-ALCL lines nor in any of the eight malignant effusions (seromas) around breast implants, although one malignant effusion contained the Th17/Th22 cytokine IL-22 [71]. Instead, four out of the eight malignant effusions contained the Th2 cytokine IL-13, and one contained IL-5. More importantly, seven out of the eight malignant effusions contained >500 pg/mL IL-9 (attributed to Th2 or, more specifically, to Th9 cells), and six out of the eight malignant effusions contained IL-10. IL-9 was not detected in any benign effusions. In contrast, IL-6 was detected in both malignant and benign peri-implant effusions [71].

### 3.5. Cytokines Modify the Microenvironment and Pathology

#### 3.5.1. BIA-ALCL

**Figure 3:**
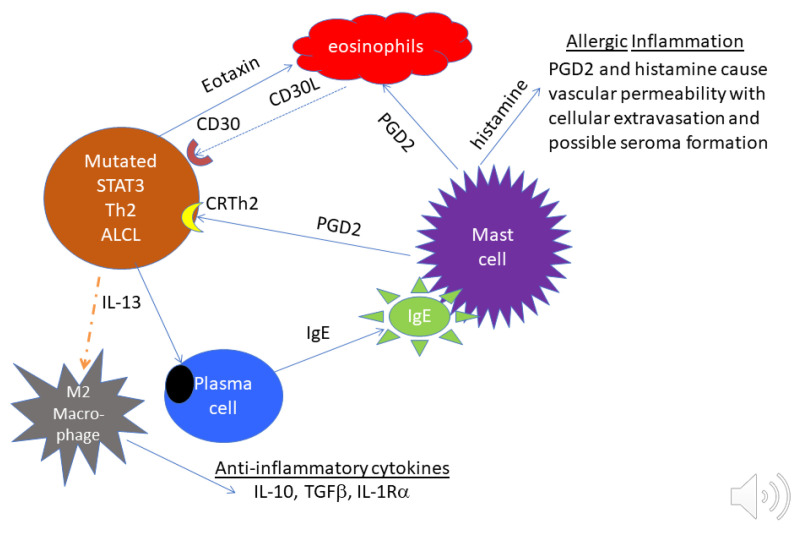
Proposed relationship between tumor cells and microenvironment in BIA-ALCL. On the left, tumor cells with mutated STAT3 release Eotaxin which attracts eosinophils. Eosinophils are known to express the CD30 ligand, which can support the proliferation of CD30+ tumor cells. At the lower left, tumor cells release IL-13 that polarizes macrophages to produce anti-inflammatory cytokines and induces plasma cells to produce IgE. In the center and upper right, IgE activates mast cells to release PGD2 to attract Th2 cells and eosinophils. PGD2 and histamine from activated mast cells cause vascular permeability, which may contribute to seroma formation.

A pathologic feature of infiltrative BIA-ALCL that distinguishes it from most systemic ALCLs is the presence of numerous eosinophils [33]. Influx of eosinophils into tissues is promoted by IL-13, Eotaxin and IL-9, which are produced by anaplastic cells in BIA-ALCL [34,71,72]. IL-13, independently and in coordination with IL-4, promotes immunoglobulin heavy chain class switching of B cells to become IgE-producing plasma cells [73]. Numerous plasma cells are often observed in capsules affected by BI-ALCL [72]. IgE binding to high-affinity receptors of mast cells (FcεR1) triggers the release of vasoactive and chemotactic factors including histamine and prostaglandin D2 (PGD2) that recruits Th2 cells, basophils and eosinophils. Indeed, one of the authors of this review (MEK) has found that anaplastic cells in BIA-ALCL often express the PGD_2_ receptor, CRTH_2_ (Figure 3). Another feature of BIA-ALCL is thickening of involved capsules due to fibrosis which can be mediated by IL-13 signaling to stimulate fibroblasts through their PDGFR [74,75].

#### 3.5.2. C-ALCL and CD30+ CLPD

Cytokines released by anaplastic cells in cutaneous lymphomas can dramatically affect the growth of keratinocytes. A noteworthy feature of CD30+ CLPD is pseudoepitheliomatous hyperplasia (PEH) of keratinocytes which can be mistaken for squamous carcinoma (Figure 1). In a study of 25 patients, two patterns of PEH were noted: (1) a follicular pattern was observed in 14 cases, commonly associated with a neutrophilic-rich infiltrate (*p* = 0.21), and (2) an epidermal pattern was observed in 11 cases and commonly associated with eosinophil-rich infiltrates (*p* = 0.03) [76]. PEH in CD30+CLPD was associated with Th17/Th22 cytokine expression detected in tumor cells in 81% of cases tested. All 14 cases tested had a strong expression of cytokeratin 17 (CK17), a myoepithelial keratin not found in a healthy epidermis, but this was induced in a dose-dependent manner by IL-17 through the signal transducer and activators of transcription STAT1 and STAT3 [76,77]. IL-22 inhibits the maturation of keratinocytes and stimulates their migration, causing epidermal remodeling and often leading to psoriasiform/PEH-like lesions [78,79,80]. Transgenic overexpression of IL-22 in mice results in psoriasis-like skin alterations including hyperproliferation, acanthosis and hypogranularity. IL-22 is produced by Th17 cells and, in a more restricted manner, by Th22 cells. While PEH lesions were associated with spontaneous regression and an indolent course, some patients developed a generalized process with tumor progression [76].

SATB1, a thymocyte-specific chromatin organizer, helps to classify CD30+ CLPDs with different clinicopathological behaviors. SATB1 expression was identified in CD30+ anaplastic T cells in 11 of 12 (91.7%) lymphomatoid papulosis cases and in 16 of 42 (38.1%) C-ALCL cases. SATB1+ cases showed Th17 polarization, together with more prominent epidermal hyperplasia and granulocytic infiltration, consistent with the above-mentioned PEH [81]. SATB1+ lesions responded better to combined treatment with low-dose (5–20 mg/weekly) methotrexate and interferon γ2b. In clinical samples of C-ALCLs, genes of the IL-13 signaling pathway, including IL13, IL13Rα1, IL13Rα2 and IL4Rα, were enriched in SATB1+ cases, compared to SATB1- cases [82]. All SATB1+ CD30+ CLPDs were found to highly express pSTAT6, and most were IL-13+. A specific inhibitor of pSTAT6 (AS1517499) reduced cell viability in Mac1/2A cutaneous ALCL cell lines in a dose-dependent manner, as described previously for Sézary cells [82], indicating that blocking the IL-13/STAT6 signaling pathway may be a potential therapeutic regimen for SATB1+ CALCLs. In systemic ALCLs, ALK+ ALCLs strongly express SATB1, while ALK-negative cases lack SATB1, and STAT3, RORC and IL17A were highly expressed in SATB1+ cases [83]. 

### 3.6. Does Cytokine Profile Indicate the Cell of Origin or Maturation Stage of ALCLs?

#### 3.6.1. Systemic ALCL

Several pieces of evidence suggest that tumor cells of systemic ALK+ ALCL display features of Th17 and/or ILC3 innate lymphoid cells. Schleussner et al. suggested some ALCLs could derive from innate lymphoid cells type 3 (ILC3) [84]. They demonstrated constitutive activation of AP-1 and IRF-4 in both ALK+ and ALK- ALCLs with AP-1 motifs bound to BATF and BATF3. The gene expression profile of ALCL cells included Th17/group 3 innate lymphoid cell (ILC3)-associated marker genes such as AHR, IL17F, IL-22, IL-26, IL-23R and RORγt. Elevated IL-17A and IL-17F plasma levels were detected in a subset of children and adolescents with ALK+ ALCL, supporting the proposed Th17/ILC3 phenotype for ALK+ systemic ALCL.

Matsuyama et al. demonstrated that NPM-ALK promoted expression of miR-135b and its host gene LEMD1 through the action of STAT3. Further, miR-135b suppressed Th2 regulators GATA3 and STAT6, and miR-153b blockade attenuated IL-17 production, leading to an ALCL phenotype overlapping with Th17 cells [85]. ALK+ ALCL cell lines express Th17-associated signature genes, including IL-17F, IL-22, IL-26, AHR and RORC [64]. 

Eckerle et al. performed gene expression profiling of microdissected lymphoma cells of five ALK(+) and four ALK(-) systemic ALCLs, seven cALCLs and sixteen cHLs, and of eight subsets of normal T and NK cells [83]. All ALCL types showed significant expression of NFkappaB target genes and upregulation of genes involved in oncogenesis (e.g., EZH2). 

Knörr et al. asked whether a Th subset-specific serum cytokine pattern could be identified in childhood ALCL patients [65]. Although some patients showed a pattern of elevated IFN-γ, IP-10 and MIG (both produced upon stimulation with IFN-γ), and levels of IL-17 and IL-23 suggested activation of Th17 cells, ALCLs of most patients did not show a conclusive Th subset pattern. 

#### 3.6.2. BIA-ALCL

The cell lineage or differentiation stage of BIA-ALCL appears to be variable. In cell lines derived from malignant seromas, two of four co-expressed the Th2 cytokine IL-13 and the Th1 cytokine IFNγ [71]. None expressed Th17 cytokines characteristic of ALK+ ALCL. Three of four cell lines secreted IL-9, which was detected in eight out of nine malignant effusions. IL-9 was originally assigned to Th2 cells but more recently to Th9 cells, which also produce IL-10, detected together with IL-9 in four out of eight malignant effusions. Such evidence points to a Th2-type cytokine profile with expression of IL-10, IL-13, IL-9 and Eotaxin and frequent expression of the GATA3 and FoxP3 transcription factors. Breast implants may elicit a Th2-type response with accumulation of T cells, mast cells and eosinophils, and, due to activation of the STAT3 pathway, IL-10-producing T regulatory cells are recruited or induced [34]. The plasticity between Th2 cells and iTregs is in accordance with such observation [86]. Di Napoli used gene expression profiling and immunohistochemical data to suggest either activation-induced FoxP3 expression or a T helper-like regulatory T cell status in a proportion of BIA-ALCLs with upregulation of the *RORC* and *IL17A* genes and of the FOXP3 protein [87]. 

Some CD30+ CLPDs are derived from a novel subset of CD4+Th2 cells that produce inflammatory Th17 cytokines [88]. This subset of Th2 cells was reported to promote exacerbation of chronic allergic asthma [89]. These results are consistent with our recent report linking atopy to the pathogenesis of lymphomatoid papulosis [90].

## 4. Therapy of ALCLs

### 4.1. Front-Line Treatments

In adult systemic ALCL, several combined chemotherapy regimens obtain a high event-free survival rate, especially for local or regional disease [19,21,91]. The addition of etoposide improved prognosis, with a 3-year survival of 100% [92]. However, relapse occurred in 20–30% of patients, requiring reinduction by high-dose chemotherapies before autologous or allogenic hematopoietic stem cell transplantation [21,91].

In pediatric patients, adapted protocols with reduced cumulative doses of toxic drugs obtained a 92% overall survival at 2 years. A risk-stratified strategy restricted autologous HCST for pediatric ALCL high-risk relapses and vinblastine for intermediate or low-risk relapses [19,93].

C-ALCL can be cured by complete surgical excision and/or local radiotherapy as first-line treatment [94]. For multifocal lesions, low-dose methotrexate is the first-line treatment, and vinblastine was also proposed at relapse [95,96].

About 80% of BIA-ALCL patients are cured by surgical resection of implants and the surrounding capsule. Adjuvant localized radiation is performed when excision is incomplete. For patients with tumors and/or regional lymph node involvement, adjuvant chemotherapy or targeted therapy is available [97].

### 4.2. Targeted Therapies

Patients with advanced or refractory/relapsing ALCL, BI-ALCL or CALCL obtain their first benefit from targeted therapies directed against CD30, ALK, NOTCH1 or JAK (Table 1 and Table 2). Some of them are now also used in combination therapy in front-line treatment [97,98,99]. 

The anti-CD30 brentuximab-vedotin is a monoclonal anti-CD30 antibody fused to a microtubule inhibitor. The latter is delivered after endocytosis and lysosome fusion (Figure 2) [99,100]. It has been approved by US and European agencies for the treatment of relapsing systemic ALCL or C-ALCL either as a single-agent therapy or in combination with chemotherapy [99,101]. The limited durability of response together with significant toxicity including neuropathy and cytopenia has led to considering other therapies such as anti-CD30 CAR T cells [102]. 

A new anti-CD158k antibody primarily developed for the treatment of Sézary syndrome can also be employed in C-ALCL [103,104]. 

ALK inhibition by first-generation inhibitor crizotinib provided promising results, especially in pediatric ALK+ ALCL, with an objective response rate ranging from 54% to 90% [19]. Resistance to ALK inhibition may be acquired by the emergence of specific ALK mutations that may be counteracted by new-generation inhibitors such as alectinib or ceritinib [105]. IL-10 autocrine synthesis and aberrant upregulation of the IL-10 receptor subunit also bypass NPM-ALK inhibition and contribute to single-ALK inhibitor resistance [66]. 

Given the resistance mechanisms arising after single-agent therapies, combination therapy associating chemotherapy with either anti-CD30 targeting or ALK inhibition is proposed for patients with relapsing disease [106].

Blocking the IL-9/Jak3 pathway has potential for treatment of both ALK+ ALCL and BIA-ALCL where tumor cells express IL-9 in an autocrine loop [71,107]. 

In a model of alternative activation of NF-kB in ALCL revealed by CRISPR screening, Wang et al found that in NF-kB-inducing kinase (NIK)-positive ALK- ALCL cells, common JAK/STAT3 mutations promote transcriptional activity of STAT3 which directly regulates NFKB2 and CD30 expression [108]. Endogenous expression of CD30 induced constitutive NF-κB activation through binding and degrading of TRAF3. In ALK+ ALCL, the CD30 pathway is blocked by NPM-ALK oncoprotein, but STAT3 activity and resultant NFKB2 expression can still be induced by NPM-ALK, leading to minimal alternative NF-κB activation. The study suggests combined NIK and JAK inhibitor therapy could benefit patients with NIK-positive ALK- ALCL carrying JAK/STAT3 somatic mutations [108]. 

ALK+ ALCL expresses a high level of PD-L1 because of the constitutive activation of multiple oncogenic signaling pathways downstream of ALK activity. In a novel model using CRISPR screening, Zhang et al discovered that PD-L1 induction was dependent on NPM-ALK activation of STAT3, as well as a signalosome containing GRB2/SOS1, which activates the MEK-ERK and PI3K-AKT signaling pathways [109]. These signaling networks, through STAT3 and the GRB2/SOS1, induce PD-L1 expression through the action of transcription factors IRF4 and BATF3 on the enhancer region of the *PD-L1* gene. IRF4 and BATF3 are essential for PD-L1 upregulation, and IRF4 expression was correlated with PD-L1 levels in primary ALK+ ALCL tissues. Targeting this oncogenic signaling pathway in ALK+ ALCL largely inhibited the ability of PD-L1-mediated tumor immune escape when cocultured with PD-1-positive T cells and natural killer cells. 

In preclinical models of ALK- ALCL, JAK 1/2 inhibition by ruxolitinib proved to be more efficient than STAT3 inhibitors [52]. Targeting the Th2 signaling pathway with a specific pSTAT6 inhibitor, AS1517499, also has potential for the treatment of C-ALCL and other CD30+ CTCLs [82,88]. Recent whole-genome sequencing of 12 C-ALCLs also underscored the potential value of PI3K-AKT inhibition for patients who are resistant to skin-directed therapy and/or with extracutaneous progression [110].

## 5. Conclusions

The four main types of ALCL differ according to the age of patients, site of onset, prognosis, tumor cell phenotype and genetic lesions. 

Translational studies have revealed key cytokine pathways and epigenetic modification regulating tumor cell growth.

Besides directly targeting tumor cells by cytotoxic drugs or antibodies, cytokine pathways may represent potential targets for personalized therapy according to the specific profile of each ALCL case.

## Figures and Tables

**Table 1:** Clinicopathological features of systemic, cutaneous and breast implant-associated anaplastic large cell lymphomas (systemic ALCL, C-ALCL, BIA-ALCL). ALK+ ALCL correspond to ALCL with *anaplastic lymphoma kinase* rearrangement.

Categories	Systemic ALK+ ALCL	Systemic ALK- ALCL	Cutaneous ALCL	BIA-ALCL
Patient age/gender	Children/adolescents	Adults	Elderly males	Women
Prognosis	Good	Poor	Excellent	Excellent
Treatment	Multiagent chemotherapy	Brentuximab-vedotin	Surgery, radiation, methotrexate	Capsulectomy
Extent of disease	Lymph nodes and frequent extranodal disease	Widespread	Skin with/without regional lymph nodes	Localized to breast
Pathology	Sheets of anaplastic cells, fibrosis in Hodgkin-like variant	Sheets of anaplastic cells	Sheets of anaplastic cells, frequent neutrophils	Individual and clusters of anaplastic cells often at capsule surface, frequent eosinophils

**Table 2:** Predominant features of the different anaplastic large cell lymphoma subtypes. ALCL, anaplastic large cell lymphoma. BIA-ALCL, breast implant-associated ALCL. * Exceptional ALK+ cutaneous ALCLs exist.

ALCL Subtype	Predominant Phenotype	Differentiation	Gene Rearrangement	Mutations	Signaling Pathways	Therapeutic Targets
Systemic ALK+ ALCL	CD30+ **ALK+, EMA+,** CD3+, CD4+, CD3-CD4-CD5-**Cytotoxic**	T-cytotoxic Th17/Th22,	***ALK*with** *NPM1* 80%		**STAT3**	**CD30, ALK, JAK**
Systemic ALK- ALCL	CD30+ **ALK-**, CD3+, CD4+, CD3-CD4-CD5-, **Cytotoxic**	T-cytotoxic	*TP63* (12.5%) *DUSP22* rare	***JAK-STAT***	**STAT3** ** ERBB4**	**CD30, JAK, HER**
Cutaneous ALCL	CD30+, **ALK-**, EMA-, CD4+, CD2-CD3-CD5-, CLA+, **Cytotoxic** **LEF1**	Th2 /Th17	***DUSP22* (30%)** *TP63* (10%) *ALK* * exceptional	***MSC*^E116K^***DUSP22* rare	**CD30-IRF4-MYC** Th2 signaling	**CD30** **pSTAT6**
		Th2	*NPM-TYK2*	*TYK2*	**STAT1** **STAT6**	**TYK**
BIA-ALCL	CD30+, **ALK-**, EMA+, TCR- CD4+ > CD8+ **Cytotoxic**	Th2	-	***JAK-STAT* and epigenetic modifiers**	**STAT3** **STAT6**	**CD30, JAK** **pSTAT6**

## References

[1] Laurent C., Baron M., Amara N., Haioun C., Dandoit M., Maynadié M., Parrens M., Vergier B., Copie-Bergman C., Fabiani B. (2017). Impact of Expert Pathologic Review of Lymphoma Diagnosis: Study of Patients From the French Lymphopath Network. J. Clin. Oncol..

[2] Parrilla Castellar E.R., Jaffe E.S., Said J.W., Swerdlow S.H., Ketterling R.P., Knudson R.A., Sidhu J.S., Hsi E.D., Karikehalli S., Jiang L. (2014). ALK-Negative Anaplastic Large Cell Lymphoma Is a Genetically Heterogeneous Disease with Widely Disparate Clinical Outcomes. Blood.

[3] Schrader A.M.R., Chung Y.-Y., Jansen P.M., Szuhai K., Bastidas Torres A.N., Tensen C.P., Willemze R. (2016). No TP63 Rearrangements in a Selected Group of Primary Cutaneous CD30+ Lymphoproliferative Disorders with Aggressive Clinical Course. Blood.

[4] Stein H., Foss H.-D., Dürkop H., Marafioti T., Delsol G., Pulford K., Pileri S., Falini B. (2000). CD30+ Anaplastic Large Cell Lymphoma: A Review of Its Histopathologic, Genetic, and Clinical Features. Blood.

[5] Feldman A.L., Law M., Remstein E.D., Macon W.R., Erickson L.A., Grogg K.L., Kurtin P.J., Dogan A. (2009). Recurrent Translocations Involving the IRF4 Oncogene Locus in Peripheral T-Cell Lymphomas. Leukemia.

[6] Pham-Ledard A., Prochazkova-Carlotti M., Laharanne E., Vergier B., Jouary T., Beylot-Barry M., Merlio J.-P. (2010). IRF4 Gene Rearrangements Define a Subgroup of CD30-Positive Cutaneous T-Cell Lymphoma: A Study of 54 Cases. J. Investig. Dermatol..

[7] Pedersen M.B., Hamilton-Dutoit S.J., Bendix K., Ketterling R.P., Bedroske P.P., Luoma I.M., Sattler C.A., Boddicker R.L., Bennani N.N., Nørgaard P. (2017). DUSP22 and TP63 Rearrangements Predict Outcome of ALK-Negative Anaplastic Large Cell Lymphoma: A Danish Cohort Study. J. Am. Soc. Hematol..

[8] DeCoteau J.F., Butmarc J.R., Kinney M.C., Kadin M.E. (1996). The t(2;5) Chromosomal Translocation Is Not a Common Feature of Primary Cutaneous CD30+ Lymphoproliferative Disorders: Comparison With Anaplastic Large-Cell Lymphoma of Nodal Origin. Blood.

[9] Beylot-Barry M., Groppi A., Vergier B., Pulford K., Merlio J.P. (1998). Characterization of t(2;5) Reciprocal Transcripts and Genomic Breakpoints in CD30+ Cutaneous Lymphoproliferations. Blood.

[10] Kadin M.E. (2006). Pathobiology of CD30+ Cutaneous T-Cell Lymphomas. J. Cutan. Pathol..

[11] Bekkenk M.W., Geelen F.A., van Voorst Vader P.C., Heule F., Geerts M.L., van Vloten W.A., Meijer C.J., Willemze R. (2000). Primary and Secondary Cutaneous CD30(+) Lymphoproliferative Disorders: A Report from the Dutch Cutaneous Lymphoma Group on the Long-Term Follow-up Data of 219 Patients and Guidelines for Diagnosis and Treatment. Blood.

[12] Beljaards R.C., Kaudewitz P., Berti E., Gianotti R., Neumann C., Rosso R., Paulli M., Meijer C.J., Willemze R. (1993). Primary Cutaneous CD30-Positive Large Cell Lymphoma: Definition of a New Type of Cutaneous Lymphoma with a Favorable Prognosis. A European Multicenter Study of 47 Patients. Cancer.

[13] Tsuyama N., Sakamoto K., Sakata S., Dobashi A., Takeuchi K. (2017). Anaplastic Large Cell Lymphoma: Pathology, Genetics, and Clinical Aspects. J. Clin. Exp. Hematopathol..

[14] Vega F., Medeiros L.J. (2020). A Suggested Immunohistochemical Algorithm for the Classification of T-Cell Lymphomas Involving Lymph Nodes. Hum. Pathol..

[15] Falini B., Pulford K., Pucciarini A., Carbone A., Wolf-Peeters C.D., Cordell J., Fizzotti M., Santucci A., Pelicci P.-G., Pileri S. (1999). Lymphomas Expressing ALK Fusion Protein(s) Other Than NPM-ALK. Blood.

[16] Turner S.D., Lamant L., Kenner L., Brugières L. (2016). Anaplastic Large Cell Lymphoma in Paediatric and Young Adult Patients. Br. J. Haematol..

[17] Boi M., Zucca E., Inghirami G., Bertoni F. (2015). Advances in Understanding the Pathogenesis of Systemic Anaplastic Large Cell Lymphomas. Br. J. Haematol..

[18] Falini B., Mason D.Y. (2002). Proteins Encoded by Genes Involved in Chromosomal Alterations in Lymphoma and Leukemia: Clinical Value of Their Detection by Immunocytochemistry. Blood.

[19] Brugieres L., Bruneau J. (2017). Anaplastic Large-Cell Lymphoma and Peripheral T-Cell Lymphoma: What Can Pediatricians and Adult Oncologists Learn from Each Other?. Hematol. Oncol..

[20] Quelen C., Grand D., Sarot E., Brugières L., Sibon D., Pradines A., Laurent C., Brousset P., Lamant L. (2020). Minimal Residual Disease Monitoring Using a 3’ALK Universal Probe Assay in ALK-Positive Anaplastic Large Cell Lymphoma: DdPCR, an Attractive Alternative Method to Real-Time Quantitative PCR. J. Mol. Diagn..

[21] Savage K.J., Harris N.L., Vose J.M., Ullrich F., Jaffe E.S., Connors J.M., Rimsza L., Pileri S.A., Chhanabhai M., Gascoyne R.D. (2008). ALK- Anaplastic Large-Cell Lymphoma Is Clinically and Immunophenotypically Different from Both ALK+ ALCL and Peripheral T-Cell Lymphoma, Not Otherwise Specified: Report from the International Peripheral T-Cell Lymphoma Project. Blood.

[22] Lamant L., de Reyniès A., Duplantier M.-M., Rickman D.S., Sabourdy F., Giuriato S., Brugières L., Gaulard P., Espinos E., Delsol G. (2007). Gene-Expression Profiling of Systemic Anaplastic Large-Cell Lymphoma Reveals Differences Based on ALK Status and Two Distinct Morphologic ALK+ Subtypes. Blood.

[23] Yamashita T., Higashi M., Momose S., Adachi A., Watanabe T., Tanaka Y., Tokuhira M., Kizaki M., Tamaru J. (2020). Decreased MYC-Associated Factor X (MAX) Expression Is a New Potential Biomarker for Adverse Prognosis in Anaplastic Large Cell Lymphoma. Sci. Rep..

[24] Lyapichev K.A., Tang G., Li S., You M.J., Cheng T.J., Miranda R.N., Iyer S., Yin C.C., Konoplev S., Bueso-Ramos C. (2021). MYC Expression Is Associated with Older Age, Common Morphology, Increased MYC Copy Number, and Poorer Prognosis in Patients with ALK+ Anaplastic Large Cell Lymphoma. Hum. Pathol..

[25] Drieux F., Ruminy P., Abdel-Sater A., Lemonnier F., Viailly P.-J., Fataccioli V., Marchand V., Bisig B., Letourneau A., Parrens M. (2020). Defining Signatures of Peripheral T-Cell Lymphoma with a Targeted 20-Marker Gene Expression Profiling Assay. Haematologica.

[26] Vasmatzis G., Johnson S.H., Knudson R.A., Ketterling R.P., Braggio E., Fonseca R., Viswanatha D.S., Law M.E., Kip N.S., Özsan N. (2012). Genome-Wide Analysis Reveals Recurrent Structural Abnormalities of TP63 and Other P53-Related Genes in Peripheral T-Cell Lymphomas. Blood.

[27] Willemze R., Cerroni L., Kempf W., Berti E., Facchetti F., Swerdlow S.H., Jaffe E.S. (2019). The 2018 Update of the WHO-EORTC Classification for Primary Cutaneous Lymphomas. Blood.

[28] Willemze R., Jaffe E.S., Burg G., Cerroni L., Berti E., Swerdlow S.H., Ralfkiaer E., Chimenti S., Diaz-Perez J.L., Duncan L.M. (2005). WHO-EORTC Classification for Cutaneous Lymphomas. Blood.

[29] Prieto-Torres L., Rodriguez-Pinilla S.M., Onaindia A., Ara M., Requena L., Piris M.Á. (2019). CD30-Positive Primary Cutaneous Lymphoproliferative Disorders: Molecular Alterations and Targeted Therapies. Haematologica.

[30] King R.L., Dao L.N., McPhail E.D., Jaffe E.S., Said J., Swerdlow S.H., Sattler C.A., Ketterling R.P., Sidhu J.S., Hsi E.D. (2016). Morphologic Features of ALK-Negative Anaplastic Large Cell Lymphomas With DUSP22 Rearrangements. Am. J. Surg. Pathol..

[31] Ravindran A.M., Feldman A.L., Ketterling R.P., Dasari S., Rech K.L., McPhail E.D., Kurtin P.J., Shi M. (2020). Striking Association of Lymphoid Enhancing Factor (LEF1) Overexpression and DUSP22 Rearrangements in Anaplastic Large Cell Lymphoma. J. Surg. Pathol..

[32] Velusamy T., Kiel M.J., Sahasrabuddhe A.A., Rolland D., Dixon C.A., Bailey N.G., Betz B.L., Brown N.A., Hristov A.C., Wilcox R.A. (2014). A Novel Recurrent NPM1-TYK2 Gene Fusion in Cutaneous CD30-Positive Lymphoproliferative Disorders. Blood.

[33] Laurent C., Delas A., Gaulard P., Haioun C., Moreau A., Xerri L., Traverse-Glehen A., Rousset T., Quintin-Roue I., Petrella T. (2016). Breast Implant-Associated Anaplastic Large Cell Lymphoma: Two Distinct Clinicopathological Variants with Different Outcomes. Ann. Oncol..

[34] Di Napoli A., Greco D., Scafetta G., Ascenzi F., Gulino A., Aurisicchio L., Santanelli Di Pompeo F., Bonifacino A., Giarnieri E., Morgan J. (2020). IL-10, IL-13, Eotaxin and IL-10/IL-6 Ratio Distinguish Breast Implant-Associated Anaplastic Large-Cell Lymphoma from All Types of Benign Late Seromas. Cancer Immunol. Immunother..

[35] Hu H., Johani K., Almatroudi A., Vickery K., Van Natta B., Kadin M.E., Brody G., Clemens M., Cheah C.Y., Lade S. (2016). Bacterial Biofilm Infection Detected in Breast Implant-Associated Anaplastic Large-Cell Lymphoma. Plast Reconstr. Surg..

[36] Laurent C., Lopez C., Desjobert C., Berrebi A., Damm-Welk C., Delsol G., Brousset P., Lamant L. (2012). Circulating t(2;5)-Positive Cells Can Be Detected in Cord Blood of Healthy Newborns. Leukemia.

[37] Malcolm T.I.M., Villarese P., Fairbairn C.J., Lamant L., Trinquand A., Hook C.E., Burke G.A.A., Brugières L., Hughes K., Payet D. (2016). Anaplastic Large Cell Lymphoma Arises in Thymocytes and Requires Transient TCR Expression for Thymic Egress. Nat. Commun..

[38] Marzec M., Halasa K., Liu X., Wang H.Y., Cheng M., Baldwin D., Tobias J.W., Schuster S.J., Woetmann A., Zhang Q. (2013). Malignant Transformation of CD4^+^ T Lymphocytes Mediated by Oncogenic Kinase NPM/ALK Recapitulates IL-2–Induced Cell Signaling and Gene Expression Reprogramming. J. Immunol..

[39] Congras A., Hoareau-Aveilla C., Caillet N., Tosolini M., Villarese P., Cieslak A., Rodriguez L., Asnafi V., Macintyre E., Egger G. (2020). ALK-Transformed Mature T Lymphocytes Restore Early Thymus Progenitor Features. J. Clin. Investig..

[40] Ellis T.M., Simms P.E., Slivnick D.J., Jäck H.M., Fisher R.I. (1993). CD30 Is a Signal-Transducing Molecule That Defines a Subset of Human Activated CD45RO+ T Cells. J. Immunol..

[41] Stein H., Mason D.Y., Gerdes J., O’Connor N., Wainscoat J., Pallesen G., Gatter K., Falini B., Delsol G., Lemke H. (1985). The Expression of the Hodgkin’s Disease Associated Antigen Ki-1 in Reactive and Neoplastic Lymphoid Tissue: Evidence That Reed-Sternberg Cells and Histiocytic Malignancies Are Derived from Activated Lymphoid Cells. Blood.

[42] Chiarle R., Voena C., Ambrogio C., Piva R., Inghirami G. (2008). The Anaplastic Lymphoma Kinase in the Pathogenesis of Cancer. Nat. Rev. Cancer.

[43] Morris S., Kirstein M., Valentine M., Dittmer K., Shapiro D., Saltman D., Look A. (1994). Fusion of a Kinase Gene, ALK, to a Nucleolar Protein Gene, NPM, in Non-Hodgkin’s Lymphoma. Science.

[44] Werner M.T., Zhao C., Zhang Q., Wasik M.A. (2017). Nucleophosmin-Anaplastic Lymphoma Kinase: The Ultimate Oncogene and Therapeutic Target. Blood.

[45] Luchtel R.A., Zimmermann M.T., Hu G., Dasari S., Jiang M., Oishi N., Jacobs H.K., Zeng Y., Hundal T., Rech K.L. (2019). Recurrent MSCE116K Mutations in ALK-Negative Anaplastic Large Cell Lymphoma. Blood.

[46] Ambrogio C., Martinengo C., Voena C., Tondat F., Riera L., di Celle P.F., Inghirami G., Chiarle R. (2009). NPM-ALK Oncogenic Tyrosine Kinase Controls T-Cell Identity by Transcriptional Regulation and Epigenetic Silencing in Lymphoma Cells. Cancer Res..

[47] Larose H., Prokoph N., Matthews J.D., Schlederer M., Högler S., Alsulami A.F., Ducray S.P., Nuglozeh E., Fazaludeen F.M.S., Elmouna A. (2021). Whole Exome Sequencing Reveals NOTCH1 Mutations in Anaplastic Large Cell Lymphoma and Points to Notch Both as a Key Pathway and a Potential Therapeutic Target. Haematologica.

[48] Merkel O., Hamacher F., Laimer D., Sifft E., Trajanoski Z., Scheideler M., Egger G., Hassler M.R., Thallinger C., Schmatz A. (2010). Identification of Differential and Functionally Active MiRNAs in Both Anaplastic Lymphoma Kinase (ALK)+ and ALK- Anaplastic Large-Cell Lymphoma. Proc. Natl. Acad. Sci. USA.

[49] Desjobert C., Renalier M.-H., Bergalet J., Dejean E., Joseph N., Kruczynski A., Soulier J., Espinos E., Meggetto F., Cavaillé J. (2011). MiR-29a down-Regulation in ALK-Positive Anaplastic Large Cell Lymphomas Contributes to Apoptosis Blockade through MCL-1 Overexpression. Blood.

[50] Crescenzo R., Abate F., Lasorsa E., Tabbo’ F., Gaudiano M., Chiesa N., Di Giacomo F., Spaccarotella E., Barbarossa L., Ercole E. (2015). Convergent Mutations and Kinase Fusions Lead to Oncogenic STAT3 Activation in Anaplastic Large Cell Lymphoma. Cancer Cell.

[51] Andersson E.I., Brück O., Braun T., Mannisto S., Saikko L., Lagström S., Ellonen P., Leppä S., Herling M., Kovanen P.E. (2020). STAT3 Mutation Is Associated with STAT3 Activation in CD30+ ALK− ALCL. Cancers.

[52] Chen J., Zhang Y., Petrus M.N., Xiao W., Nicolae A., Raffeld M., Pittaluga S., Bamford R.N., Nakagawa M., Ouyang S.T. (2017). Cytokine Receptor Signaling Is Required for the Survival of ALK− Anaplastic Large Cell Lymphoma, Even in the Presence of JAK1/STAT3 Mutations. Proc. Natl. Acad. Sci. USA.

[53] Laurent C., Nicolae A., Laurent C., Le Bras F., Haioun C., Fataccioli V., Amara N., Adélaïde J., Guille A., Schiano J.-M. (2020). Gene Alterations in Epigenetic Modifiers and JAK-STAT Signaling Are Frequent in Breast Implant–Associated ALCL. Blood.

[54] Mélard P., Idrissi Y., Andrique L., Poglio S., Prochazkova-Carlotti M., Berhouet S., Boucher C., Laharanne E., Chevret E., Pham-Ledard A. (2016). Molecular Alterations and Tumor Suppressive Function of the *DUSP22* (Dual Specificity Phosphatase 22) Gene in Peripheral T-Cell Lymphoma Subtypes. Oncotarget.

[55] Luchtel R.A., Dasari S., Oishi N., Pedersen M.B., Hu G., Rech K.L., Ketterling R.P., Sidhu J., Wang X., Katoh R. (2018). Molecular Profiling Reveals Immunogenic Cues in Anaplastic Large Cell Lymphomas with DUSP22 Rearrangements. Blood.

[56] Scarfò I., Pellegrino E., Mereu E., Kwee I., Agnelli L., Bergaggio E., Garaffo G., Vitale N., Caputo M., Machiorlatti R. (2016). Identification of a New Subclass of ALK-Negative ALCL Expressing Aberrant Levels of ERBB4 Transcripts. Blood.

[57] Borchmann P. (2008). CD30+ Diseases: Anaplastic Large-Cell Lymphoma and Lymphomatoid Papulosis. Cancer Treat Res.

[58] Gause A., Jung W., Keymis S., Schobert I., Scholz R., Schmits R., Diehl V., Pohl C., Hasenclever D., Pfreundschuh M. (1992). The Clinical Significance of Cytokines and Soluble Forms of Membrane-Derived Activation Antigens in the Serum of Patients with Hodgkin’s Disease. Leuk. Lymphoma.

[59] Gause A., Jung W., Schmits R., Tschiersch A., Scholz R., Pohl C., Hasenclever D., Diehl V., Pfreundschuh M. (1992). Soluble CD8, CD25 and CD30 Antigens as Prognostic Markers in Patients with Untreated Hodgkin’s Lymphoma. Ann. Oncol..

[60] Gause A., Keymis S., Scholz R., Schobert I., Jung W., Diehl V., Pohl C., Pfreundschuh M. (1992). Increased Levels of Circulating Cytokines in Patients with Untreated Hodgkin’s Disease. Lymphokine Cytokine Res..

[61] Wasik M.A., Vonderheid E.C., Bigler R.D., Marti R., Lessin S.R., Polansky M., Kadin M.E. (1996). Increased Serum Concentration of the Soluble Interleukin-2 Receptor in Cutaneous T-Cell Lymphoma. Clinical and Prognostic Implications. Arch. Dermatol..

[62] Hanson S.E., Hassid V.J., Branch-Brooks C., Liu J., Kadin M.E., Miranda R., Butler C.E., Clemens M.W. (2020). Validation of a CD30 Enzyme-Linked Immunosorbant Assay for the Rapid Detection of Breast Implant-Associated Anaplastic Large Cell Lymphoma. Aesthet. Surg. J..

[63] Kadin M.E., Pavlov I., Delgado J.C., Vonderheid E.C. (2012). High Soluble CD30, CD25 and IL-6 May Identify Patients with Worse Survival in CD30+ Cutaneous Lymphomas and Early Mycosis Fungoides. J. Investig. Dermatol..

[64] Savan R., McFarland A.P., Reynolds D.A., Feigenbaum L., Ramakrishnan K., Karwan M., Shirota H., Klinman D.M., Dunleavy K., Pittaluga S. (2011). A Novel Role for IL-22R1 as a Driver of Inflammation. Blood.

[65] Knörr F., Damm-Welk C., Ruf S., Singh V.K., Zimmermann M., Reiter A., Woessmann W. (2018). Blood Cytokine Concentrations in Pediatric Patients with Anaplastic Lymphoma Kinase-Positive Anaplastic Large Cell Lymphoma. Haematologica.

[66] Prokoph N., Probst N.A., Lee L.C., Monahan J.M., Matthews J.D., Liang H.-C., Bahnsen K., Montes-Mojarro I.A., Karaca-Atabay E., Sharma G.G. (2020). IL10RA Modulates Crizotinib Sensitivity in NPM1-ALK+ Anaplastic Large Cell Lymphoma. Blood.

[67] Zhang Q., Nowak I., Vonderheid E.C., Rook A.H., Kadin M.E., Nowell P.C., Shaw L.M., Wasik M.A. (1996). Activation of Jak/STAT Proteins Involved in Signal Transduction Pathway Mediated by Receptor for Interleukin 2 in Malignant T Lymphocytes Derived from Cutaneous Anaplastic Large T-Cell Lymphoma and Sezary Syndrome. Proc. Natl. Acad. Sci. USA.

[68] Ihle J.N., Witthuhn B.A., Quelle F.W., Yamamoto K., Silvennoinen O. (1995). Signaling Through the Hematopoietic Cytokine Receptors. Annu. Rev. Immunol..

[69] Miyazaki T., Kawahara A., Fujii H., Nakagawa Y., Minami Y., Liu Z., Oishi I., Silvennoinen O., Witthuhn B., Ihle J. (1994). Functional Activation of Jak1 and Jak3 by Selective Association with IL-2 Receptor Subunits. Science.

[70] Wasik M.A., Sioutos N., Tuttle M., Butmarc J.R., Kaplan W.D. (1994). Constitutive Secretion of Soluble Interleukin-2 Receptor by Human T Cell Lymphoma Xenografted into SCID Mice. Am. J. Pathol..

[71] Kadin M.E., Morgan J., Kouttab N., Xu H., Adams W.P., Glicksman C., McGuire P., Sieber D., Epstein A.L., Miranda R.N. (2020). Comparative Analysis of Cytokines of Tumor Cell Lines, Malignant and Benign Effusions Around Breast Implants. Aesthet. Surg. J..

[72] Kadin M.E., Morgan J., Xu H., Epstein A.L., Sieber D., Hubbard B.A., Adams W.P., Bacchi C.E., Goes J.C.S., Clemens M.W. (2018). IL-13 Is Produced by Tumor Cells in Breast Implant–Associated Anaplastic Large Cell Lymphoma: Implications for Pathogenesis. Hum. Pathol..

[73] Punnonen J., Aversa G., Cocks B.G., McKenzie A.N., Menon S., Zurawski G., de Waal Malefyt R., de Vries J.E. (1993). Interleukin 13 Induces Interleukin 4-Independent IgG4 and IgE Synthesis and CD23 Expression by Human B Cells. Proc. Natl. Acad. Sci. USA.

[74] Gasch M., Goroll T., Bauer M., Hinz D., Schütze N., Polte T., Kesper D., Simon J.C., Hackermüller J., Lehmann I. (2014). Generation of IL-8 and IL-9 Producing CD4+ T Cells Is Affected by Th17 Polarizing Conditions and AHR Ligands. Mediat. Inflamm..

[75] Gieseck R.L., Ramalingam T.R., Hart K.M., Vannella K.M., Cantu D.A., Lu W.-Y., Ferreira-González S., Forbes S.J., Vallier L., Wynn T.A. (2016). Interleukin-13 Activates Distinct Cellular Pathways Leading to Ductular Reaction, Steatosis, and Fibrosis. Immunity.

[76] Guitart J., Martinez-Escala M.E., Deonizio J.M.D., Gerami P., Kadin M.E. (2015). CD30+ Cutaneous Lymphoproliferative Disorders with Pseudocarcinomatous Hyperplasia Are Associated with a T-Helper-17 Cytokine Profile and Infiltrating Granulocytes. J. Am. Acad. Dermatol..

[77] DePianto D., Kerns M.L., Dlugosz A.A., Coulombe P.A. (2010). Keratin 17 Promotes Epithelial Proliferation and Tumor Growth by Polarizing the Immune Response in Skin. Nat. Genet..

[78] Eyerich S., Eyerich K., Pennino D., Carbone T., Nasorri F., Pallotta S., Cianfarani F., Odorisio T., Traidl-Hoffmann C., Behrendt H. Th22 Cells Represent a Distinct Human T Cell Subset Involved in Epidermal Immunity and Remodeling. http://www.jci.org/articles/view/40202/pdf.

[79] Wolk K., Witte E., Wallace E., Döcke W.-D., Kunz S., Asadullah K., Volk H.-D., Sterry W., Sabat R. (2006). IL-22 Regulates the Expression of Genes Responsible for Antimicrobial Defense, Cellular Differentiation, and Mobility in Keratinocytes: A Potential Role in Psoriasis. Eur. J. Immunol..

[80] Wolk K., Haugen H.S., Xu W., Witte E., Waggie K., Anderson M., Vom Baur E., Witte K., Warszawska K., Philipp S. (2009). IL-22 and IL-20 Are Key Mediators of the Epidermal Alterations in Psoriasis While IL-17 and IFN-Gamma Are Not. J. Mol. Med. (Berl.).

[81] Sun J., Yi S., Qiu L., Fu W., Wang A., Liu F., Wang L., Wang T., Chen H., Wang L. (2018). SATB1 Defines a Subtype of Cutaneous CD30+ Lymphoproliferative Disorders Associated with a T-Helper 17 Cytokine Profile. J. Investig. Dermatol..

[82] Geskin L.J., Viragova S., Stolz D.B., Fuschiotti P. (2015). Interleukin-13 Is Overexpressed in Cutaneous T-Cell Lymphoma Cells and Regulates Their Proliferation. Blood.

[83] Eckerle S., Brune V., Döring C., Tiacci E., Bohle V., Sundström C., Kodet R., Paulli M., Falini B., Klapper W. (2009). Gene Expression Profiling of Isolated Tumour Cells from Anaplastic Large Cell Lymphomas: Insights into Its Cellular Origin, Pathogenesis and Relation to Hodgkin Lymphoma. Leukemia.

[84] Schleussner N., Merkel O., Costanza M., Liang H.-C., Hummel F., Romagnani C., Durek P., Anagnostopoulos I., Hummel M., Jöhrens K. (2018). The AP-1-BATF and -BATF3 Module Is Essential for Growth, Survival and TH17/ILC3 Skewing of Anaplastic Large Cell Lymphoma. Leukemia.

[85] Matsuyama H., Suzuki H.I., Nishimori H., Noguchi M., Yao T., Komatsu N., Mano H., Sugimoto K., Miyazono K. (2011). MiR-135b Mediates NPM-ALK–Driven Oncogenicity and Renders IL-17–Producing Immunophenotype to Anaplastic Large Cell Lymphoma. Blood.

[86] Wan Y.Y., Flavell R.A. (2007). Regulatory T-Cell Functions Are Subverted and Converted Owing to Attenuated Foxp3 Expression. Nature.

[87] Di Napoli A., De Cecco L., Piccaluga P.P., Navari M., Cancila V., Cippitelli C., Pepe G., Lopez G., Monardo F., Bianchi A. (2019). Transcriptional Analysis Distinguishes Breast Implant-Associated Anaplastic Large Cell Lymphoma from Other Peripheral T-Cell Lymphomas. Mod. Pathol..

[88] Wen Y., Sun J., Yi S., Gao Y., Kouttab N., Morgan J., Wang Y., Kadin M.E. (2020). IL-13 Signaling in CD30+ Cutaneous Lymphoproliferative Disorders. J. Investig. Dermatol..

[89] Wang Y.-H., Voo K.S., Liu B., Chen C.-Y., Uygungil B., Spoede W., Bernstein J.A., Huston D.P., Liu Y.-J. (2010). A Novel Subset of CD4+ TH2 Memory/Effector Cells That Produce Inflammatory IL-17 Cytokine and Promote the Exacerbation of Chronic Allergic Asthma. J. Exp. Med..

[90] Kadin M.E., Hamilton R.G., Vonderheid E.C. (2020). Evidence Linking Atopy and Staphylococcal Superantigens to the Pathogenesis of Lymphomatoid Papulosis, a Recurrent CD30+ Cutaneous Lymphoproliferative Disorder. PLoS ONE.

[91] Schmitz N., Trümper L., Ziepert M., Nickelsen M., Ho A.D., Metzner B., Peter N., Loeffler M., Rosenwald A., Pfreundschuh M. (2010). Treatment and Prognosis of Mature T-Cell and NK-Cell Lymphoma: An Analysis of Patients with T-Cell Lymphoma Treated in Studies of the German High-Grade Non-Hodgkin Lymphoma Study Group. Blood.

[92] Sibon D., Nguyen D.-P., Schmitz N., Suzuki R., Feldman A.L., Gressin R., Lamant L., Weisenburger D.D., Rosenwald A., Nakamura S. (2019). ALK-Positive Anaplastic Large-Cell Lymphoma in Adults: An Individual Patient Data Pooled Analysis of 263 Patients. Haematologica.

[93] Brugières L., Pacquement H., Le Deley M.-C., Leverger G., Lutz P., Paillard C., Baruchel A., Frappaz D., Nelken B., Lamant L. (2009). Single-Drug Vinblastine as Salvage Treatment for Refractory or Relapsed Anaplastic Large-Cell Lymphoma: A Report from the French Society of Pediatric Oncology. J. Clin. Oncol..

[94] Kempf W., Pfaltz K., Vermeer M.H., Cozzio A., Ortiz-Romero P.L., Bagot M., Olsen E., Kim Y.H., Dummer R., Pimpinelli N. (2011). EORTC, ISCL, and USCLC Consensus Recommendations for the Treatment of Primary Cutaneous CD30-Positive Lymphoproliferative Disorders: Lymphomatoid Papulosis and Primary Cutaneous Anaplastic Large-Cell Lymphoma*. Blood.

[95] Fujita H., Nagatani T., Miyazawa M., Wada H., Koiwa K., Komatsu H., Ikezawa Z. (2008). Primary Cutaneous Anaplastic Large Cell Lymphoma Successfully Treated with Low-Dose Oral Methotrexate. Eur. J. Dermatol..

[96] Laly P., Ingen-Housz-Oro S., Beylot-Barry M., Verneuil L., Adamski H., Brice P., Bagot M. (2015). Efficacy of Vinblastine in Primary Cutaneous Anaplastic Large Cell Lymphoma. JAMA Dermatol..

[97] Mehta-Shah N., Clemens M.W., Horwitz S.M. (2018). How I Treat Breast Implant–Associated Anaplastic Large Cell Lymphoma. Blood.

[98] Fanale M.A., Horwitz S.M., Forero-Torres A., Bartlett N.L., Advani R.H., Pro B., Chen R.W., Davies A., Illidge T., Uttarwar M. (2018). Five-Year Outcomes for Frontline Brentuximab Vedotin with CHP for CD30-Expressing Peripheral T-Cell Lymphomas. Blood.

[99] Donato E.M., Fernández-Zarzoso M., Hueso J.A., de la Rubia J. (2018). Brentuximab Vedotin in Hodgkin Lymphoma and Anaplastic Large-Cell Lymphoma: An Evidence-Based Review. OncoTargets Ther..

[100] Senter P.D., Sievers E.L. (2012). The Discovery and Development of Brentuximab Vedotin for Use in Relapsed Hodgkin Lymphoma and Systemic Anaplastic Large Cell Lymphoma. Nat. Biotechnol..

[101] Prince H.M., Kim Y.H., Horwitz S.M., Dummer R., Scarisbrick J., Quaglino P., Zinzani P.L., Wolter P., Sanches J.A., Ortiz-Romero P.L. (2017). Brentuximab Vedotin or Physician’s Choice in CD30-Positive Cutaneous T-Cell Lymphoma (ALCANZA): An International, Open-Label, Randomised, Phase 3, Multicentre Trial. Lancet.

[102] Ramos C.A., Ballard B., Zhang H., Dakhova O., Gee A.P., Mei Z., Bilgi M., Wu M.-F., Liu H., Grilley B. (2017). Clinical and Immunological Responses after CD30-Specific Chimeric Antigen Receptor–Redirected Lymphocytes. J. Clin. Investig..

[103] Bagot M., Porcu P., Marie-Cardine A., Battistella M., William B.M., Vermeer M., Whittaker S., Rotolo F., Ram-Wolff C., Khodadoust M.S. (2019). IPH4102, a First-in-Class Anti-KIR3DL2 Monoclonal Antibody, in Patients with Relapsed or Refractory Cutaneous T-Cell Lymphoma: An International, First-in-Human, Open-Label, Phase 1 Trial. Lancet Oncol..

[104] Battistella M., Janin A., Jean-Louis F., Collomb C., Leboeuf C., Sicard H., Bonnafous C., Dujardin A., Ram-Wolff C., Kadin M.E. (2016). KIR3DL2 (CD158k) Is a Potential Therapeutic Target in Primary Cutaneous Anaplastic Large-Cell Lymphoma. Br. J. Dermatol..

[105] Mossé Y.P., Voss S.D., Lim M.S., Rolland D., Minard C.G., Fox E., Adamson P., Wilner K., Blaney S.M., Weigel B.J. (2017). Targeting ALK With Crizotinib in Pediatric Anaplastic Large Cell Lymphoma and Inflammatory Myofibroblastic Tumor: A Children’s Oncology Group Study. J. Clin. Oncol..

[106] Van der Weyden C.A., Pileri S.A., Feldman A.L., Whisstock J., Prince H.M. (2017). Understanding CD30 Biology and Therapeutic Targeting: A Historical Perspective Providing Insight into Future Directions. Blood Cancer J..

[107] Qiu L., Lai R., Lin Q., Lau E., Thomazy D.M., Calame D., Ford R.J., Kwak L.W., Kirken R.A., Amin H.M. (2006). Autocrine Release of Interleukin-9 Promotes Jak3-Dependent Survival of ALK+ Anaplastic Large-Cell Lymphoma Cells. Blood.

[108] Wang H., Wei W., Zhang J.-P., Song Z., Li Y., Xiao W., Liu Y., Zeng M.-S., Petrus M.N., Thomas C.J. (2021). A Novel Model of Alternative NF-ΚB Pathway Activation in Anaplastic Large Cell Lymphoma. Leukemia.

[109] Zhang J.-P., Song Z., Wang H.-B., Lang L., Yang Y.-Z., Xiao W., Webster D.E., Wei W., Barta S.K., Kadin M.E. (2019). A Novel Model of Controlling PD-L1 Expression in ALK+ Anaplastic Large Cell Lymphoma Revealed by CRISPR Screening. Blood.

[110] Bastidas Torres A.N., Melchers R.C., Van Grieken L., Out-Luiting J.J., Mei H., Agaser C., Kuipers T.B., Quint K.D., Willemze R., Vermeer M.H. (2021). Whole-Genome Profiling of Primary Cutaneous Anaplastic Large Cell Lymphoma. Haematologica.

